# Survival Outcomes and Late Toxicity of Postoperative Radiotherapy in Patients With Adenoid Cystic Carcinoma of the External Auditory Canal

**DOI:** 10.1002/cam4.71501

**Published:** 2025-12-29

**Authors:** Li Wang, Xinmao Song, Zhenhu Li, Haojiong Zhang, Gang Yang, Tianci Tang, Lifen Zou, Zi‐Wei Tu, Xiaoshen Wang

**Affiliations:** ^1^ Department of Radiation Oncology, Eye & ENT Hospital Fudan University Shanghai People's Republic of China; ^2^ Department of Oncology, Jinshan Hospital Fudan University Shanghai People's Republic of China; ^3^ Department of Radiation Oncology, Shanghai Proton and Heavy Ion Center Fudan University Cancer Hospital Shanghai People's Republic of China; ^4^ NHC Key Laboratory of Personalized Diagnosis and Treatment of Nasopharyngeal Carcinoma, Jiangxi Cancer Hospital The Second Affiliated Hospital of Nanchang Medical College Nanchang People's Republic of China; ^5^ Department of Radiation Oncology, Jiangxi Clinical Research Center for Cancer, Jiangxi Cancer Hospital The Second Affiliated Hospital of Nanchang Medical College Nanchang People's Republic of China

**Keywords:** adenoid cystic carcinoma (ACC), distant metastasis, external auditory canal, late toxicity, radiotherapy

## Abstract

**Background:**

Adenoid cystic carcinoma (ACC) of the external auditory canal (EAC) is an extremely rare head and neck malignancy. The study aimed to investigate the long‐term outcomes and late toxicity of postoperative radiotherapy (PORT) for patients with EAC ACC.

**Methods:**

Between 2004 and 2021, 60 patients with EAC ACC were retrospectively analyzed. The overall survival (OS), local recurrence‐free survival (LRFS), nodal recurrence‐free survival (NRFS), and distant metastasis‐free survival (DMFS) were calculated by the Kaplan–Meier method.

**Results:**

With a median follow‐up of 110.3 months, the 10‐year OS, LRFS, NRFS, and DMFS rates were 84.2%, 79.5%, 96.2%, and 59.9% after PORT, respectively. Eleven patients (18.3%) experienced local failure, 2 (3.3%) had regional failure, and 23 (38.3%) had distant metastasis (DM). Multivariate analysis showed that the advanced T stage (T3–4) was an independent risk factor for OS (*p* = 0.019) and DMFS (*p* = 0.006). Late toxicities were assessed in 45 patients. Among them, 73.3% (33/45) of patients experienced grade 3–4 ipsilateral hearing loss after surgery. Furthermore, xerostomia (31.1%, 14/45) and radiation‐related caries (26.7%, 12/45) were the most common late toxicities of radiotherapy‐related. For clinical N0 (cN0) patients (*n* = 58), the ipsilateral elective neck irradiation (ENI) did not improve the OS (*p* = 0.929), NRFS (*p* = 0.317), or DMFS (*p* = 0.778) for the advanced patients (T3–4).

**Conclusions:**

Surgery plus PORT is associated with excellent long‐term survival with tolerable late toxicity for EAC ACC patients. ENI of cN0 patients shows no survival benefit, which suggests that the omission of ENI is reasonable to reduce radiation‐related toxicity.

## 1 Introduction

Adenoid cystic carcinoma (ACC) is an indolent but aggressive tumor that may arise in a wide variety of anatomical locations in the head and neck [1, 2]. ACC in the external auditory canal (EAC) is an extremely rare clinical disease, with an annual incidence of 1–6 cases per million people [3]. A few case reports or small sample size series have been published in the previous literature. Common clinical presentations include otalgia and a slow‐growing, painless mass in the EAC [4]. Due to the lack of awareness of the disease in the early stage, patients with ACC of the EAC are frequently misdiagnosed as otitis media.

Because of the frequently indolent progression, long‐term follow‐up (≥ 10 years) is crucial to evaluate the treatment mode efficacy for patients with EAC ACC. The main failure patterns of EAC ACC are local failure and/or late distant metastases (DM), especially in the lung [4, 5, 6]. The “gold‐standard” treatment mode for EAC ACC is still radical surgical resection [7]; and the application of adjuvant or definitive radiotherapy (RT) may significantly reduce local failures [8, 9, 10, 11]. However, there is little valuable data regarding the long‐term outcomes and late toxicity in patients after RT. Especially due to the low objective response rate to chemotherapy, the treatment of DM has always been a challenge [12].

The incidence rate, clinical characteristics, and survival outcomes of ACC vary according to the location of the primary tumor. As an extremely rare disease, few studies have been reported that specialized in the role of radiotherapy for EAC ACC. In this study, we explored the long‐term outcomes and incidence of late toxicity for patients with this rare disease who underwent surgery plus PORT. We aimed to investigate the role of radiation therapy (RT) in the treatment and evaluate the late toxicities after multidisciplinary treatment.

## 2 Materials and Methods

### 2.1 Patient Population

Between January 2004 and December 2021, a total of 68 consecutive patients with EAC ACC were treated in the Department of Radiation Oncology, Eye & ENT Hospital of Fudan University. The diagnosis was confirmed pathologically. Exclusion criteria were: metastatic disease; prior head and neck radiation at any time; inability to attend the full course of radiotherapy or follow‐up visits. Eventually, a total of 60 patients were included. Clinical information on patient characteristics (age and gender), imaging findings, treatment modalities, timing of radiation therapy, treatment results, and follow‐up were obtained both from hospital charts and from the office records of the physicians who treated the patients. Late toxicities related to treatment were investigated according to the questionnaire. Fifteen patients were not evaluated for late toxic effects due to death or refusal to disclose details (Figure S1). The Clinical Research Ethics Committee of the Eye, Ear, Nose & Throat Hospital of Fudan University approved this study (2021033‐1), and all the patients provided written informed consent before treatment. The Pittsburgh staging system was used to restage the disease [13].

### 2.2 Radiotherapy

Three‐dimensional conformal radiotherapy (3D‐CRT) (*n* = 44), intensity‐modulated radiotherapy (IMRT) (*n* = 8), or volumetric modulated arc therapy (VMAT) (*n* = 8) was administered in our hospital for the patients. The doses were prescribed as follows: 66–74 Gy/30–35 fractions for gross tumor volume (GTV) of the residual disease and metastatic lymph node; 58–64 Gy at 30–35 fractions for high‐risk clinical target volume (CTV); and 50–56 Gy at 30–35 fractions for low‐risk CTV. The expanding CTV defined the planning target volume (PTV) with a 3 mm margin. RT was given once daily, five fractions per week.

### 2.3 Chemotherapy

Postoperative chemotherapy (PAC) was prone to be delivered to patients with T3–4, *N*+ lesions, positive surgical margins, and vascular invasion. PAC was performed every 21 days with the following regimens: cisplatin/nedaplatin (25 mg/m^2^ on Day 1–3), cisplatin (25 mg/m^2^ on Day 1–3) + 5‐fluorouracil (750 mg/m^2^ on Day 1–5), and cisplatin (25 mg/m^2^ on Day 1–3) + cyclophosphamide (700 mg/m^2^ on Day 1) + pirarubicin (70 mg/m^2^ on Day 1).

### 2.4 Evaluation and Follow‐Up

Treatment results were assessed by complete physical examination, magnetic resonance imaging (MRI) or computed tomography (CT) scan of the ear, chest CT scan, and abdominal ultrasound scan 3 months after RT, and then every 6–12 months (as for some patients with solid type, the risk of distant metastasis is extremely high, mostly within a year). A PET/CT scan was performed to determine the distant metastasis if needed. The primary objective of this study was overall survival (OS) and distant metastasis‐free survival (DMFS). OS was defined as the time from the date of pathological diagnosis until the date of death from any cause; DMFS was defined as the time from the date of pathological diagnosis to the development of any distant metastasis. The National Cancer Institute's Common Terminology Criteria for Adverse Events (CTCAE) version 5.0 was the preferred method for determining toxicities after treatments. Late toxicity was defined as toxicities occurring at least 30 months post‐treatment.

### 2.5 Statistical Analysis

The OS, LRFS, NRFS and DMFS were calculated using Kaplan–Meier methods. Multivariate Cox regression was performed to determine the prognostic factors for OS and DMFS. Cox proportional hazards regression analysis calculated hazard ratios (HRs), confidence intervals (CI), and *p*‐values using SPSS statistical software (Version 26.0, IBM, New York, USA) and GraphPad Prism 8.0. The Fine‐Gray model was conducted in R (version 4.2.0). Statistical significance was defined as a two‐tailed *p* < 0.05.

## 3 Results

### 3.1 Patients' Characteristics

A total of 60 patients with EAC ACC who underwent RT were enrolled in this study. The most common clinical presentation was otalgia (71.7%, 43/60), followed by an EAC mass (55.0%, 33/60), hearing loss (13.3%, 8/60), otorrhea (11.7%, 7/60), ear stuffy (6.7%, 4/60), and facial palsy (6.7%, 4/60) (Table S1). Clinical characteristics of the patients are summarized in Table 1. The median age at enrollment was 50.5 years (range, 27–72 years); there were 34 male and 26 female patients in the present study; twenty (33.3%, 20/60) patients had clinical symptoms over two years before the diagnosis was established. A total of 39 (65.0%, 39/60) patients presented with advanced T (T3–4) classification disease, and two (3.3%, 2/60) patients had nodal metastasis at the time of presentation, which affected levels VIII and X. All patients underwent surgery; 42 (70.0%, 42/60) had close or positive margins in the latest surgery. Twenty‐nine (48.3%, 29/60) patients had pathological perineural invasion (PNI) at diagnosis by the histopathological observation. Thirty‐two patients (53.3%, 32/60) received a radiation dose of ≥ 66Gy (range, 66–74 Gy). For the entire cohort, nine patients (15%, 9/60) received chemotherapy. The MRI imaging (pre‐ and post‐operation) and irradiation dose map of a typical EAC ACC patient are shown in Figure S2.

**TABLE 1:** Patients' clinical characteristics (*n* = 60).

Characteristic	No. of patients	%
Age, years		
Median (range), years	50.5	
< 50 (%)	27	45.0
≥ 50 (%)	33	55.0
Gender		
Male	34	56.7
Female	26	43.3
Duration of symptoms		
< 24 months	40	66.7
≥ 24 months	20	33.3
Pittsburgh stage (T)		
T1–2	21	35.0
T3–4	39	65.0
N status (N)		
N+	2	3.3
N−	58	96.7
Distant metastasis (M)		
M0	60	100
M1	0	0
Surgical margin		
Positive	42	70.0
Negative	18	30.0
PNI		
Yes	29	48.3
No	31	51.7
RT technique		
3D‐CRT	44	73.3
IMRT/VMAT	16	16.7
RT dose		
50–66 Gy	28	46.7
≥ 66Gy	32	53.3
Fractional dose		
2.0 Gy	48	80.0
2.12–2.25 Gy	12	20.0
ENI		
With	27	45.0
Without	33	55.0
Chemotherapy		
Yes	9	15.0
No	51	85.0

Abbreviations: 3D‐CRT, three‐dimensional conformal radiotherapy; ENI, elective neck irradiation; IMRT, intensity‐modulated radiotherapy; PNI, perineural invasion; RT, radiotherapy; VMAT, volumetric modulated arc therapy.

### 3.2 Treatment Outcomes

**FIGURE 1:**
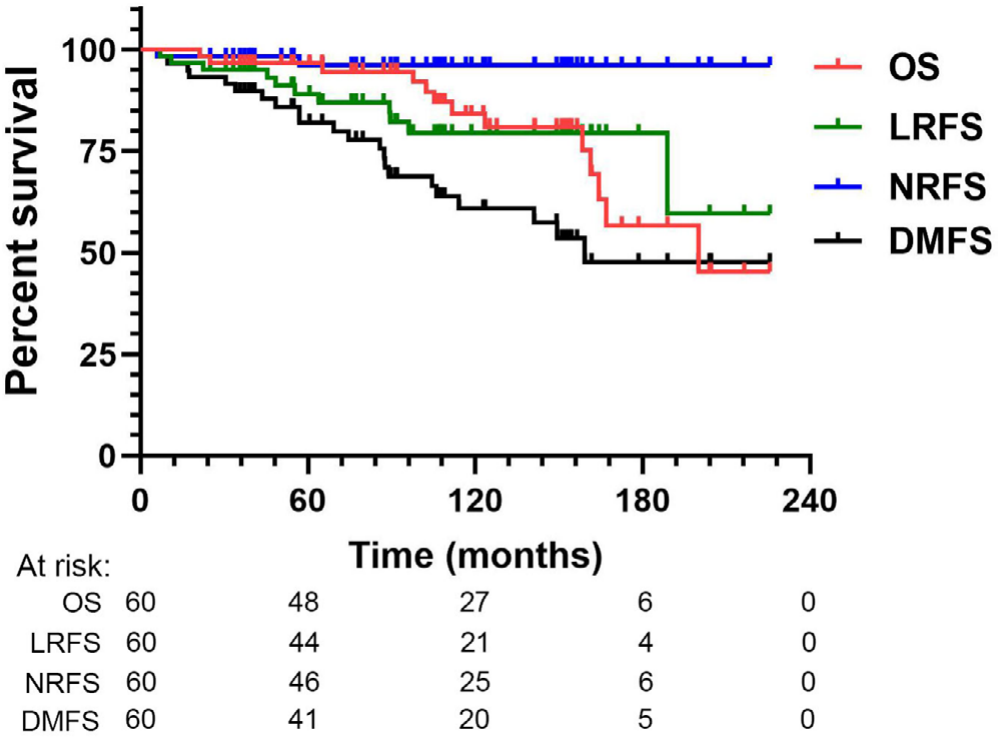
Kaplan–Meier estimates of overall survival (OS), local recurrence‐free survival (LRFS), nodal recurrence‐free survival (NRFS), and distant metastasis‐free survival (DMFS) for 60 patients with the EAC ACC treated with postoperative radiotherapy.

The median follow‐up period from diagnosis to the last visit was 110.3 months (range, 21.3–225.5 months). For the entire cohort, the 5‐, 10‐, and 15‐year OS rates were 96.7%, 84.2%, and 56.8%, respectively; the 5‐, 10‐, and 15‐year LRFS rates were 89.1%, 79.5%, and 79.5%, respectively; the 5‐, 10‐, and 15‐year NRFS rates were 96.2%, 96.2%, and 96.2%, respectively; the 5‐, 10‐, and 15‐year DMFS rates were 80.6%, 59.9%, and 46.9%, respectively (Figure 1).

**FIGURE 2:**
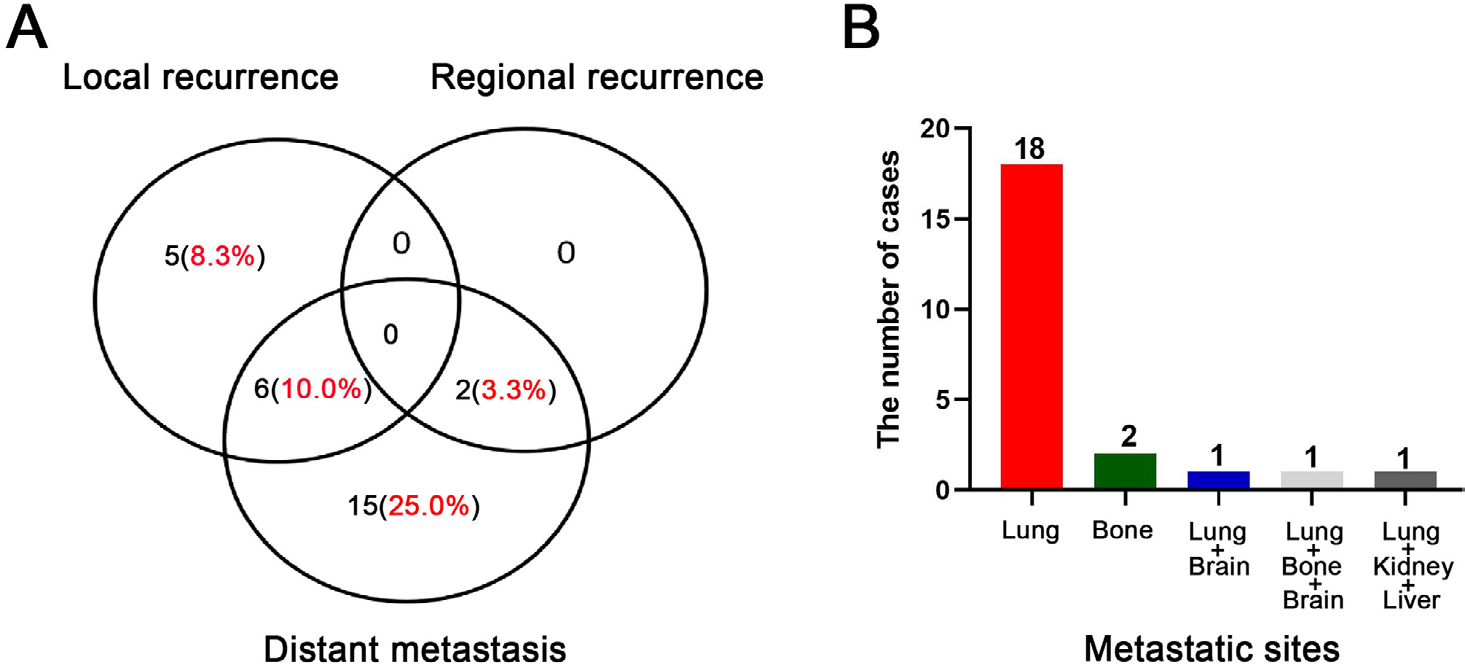
(A) Patterns of failure after radiotherapy. (B) Metastatic sites after radiotherapy.

At the time of this analysis, twenty‐eight of 60 patients (46.7%, 28/60) had treatment failures, including local recurrence (8.3%, 5/60), distant metastasis (25.0%, 15/60), or both (10.0%, 6/60) (Figure 2A); two (3.3%, 2/60) had synchronous regional recurrence and distant failure. There was one patient who experienced failure in level Ib and one in level II. Both of these two patients underwent ipsilateral ENI (one with II, III level and another with II, III, IV level). The median time from RT to local recurrence was 45.8 months (range, 3.9–188.3 months), and to distant metastasis was 42.9 months (range, 0.7–148.2 months), respectively; the median survival time after local recurrence and after distant metastasis was 77.6 months (range, 2.0–150.0 months) and 41.7 months (range, 2.0–107.5 months), respectively. Among the 23 patients with distant failure, the lung was the most predominant site of distant metastasis (*n* = 21), followed by the bone (*n* = 3); one had multiple metastases to the lung, kidney, and liver; and another one had multiple metastases to the lung, bone, and brain (Figures 2B and S3).

Thirteen patients (21.7%, 13/60) died from their disease during follow‐up. Of these, ten patients died from distant metastasis, and three died from local recurrence. The median time from diagnosis of metastasis to death was 36.1 months (range from 10.1 to 107.5 months), and the time from local recurrence to death for the three patients was 13.2, 78.1, and 103.9 months, respectively. Table S2 shows the clinical features and the cause of death analysis of the 13 patients.

**FIGURE 3:**
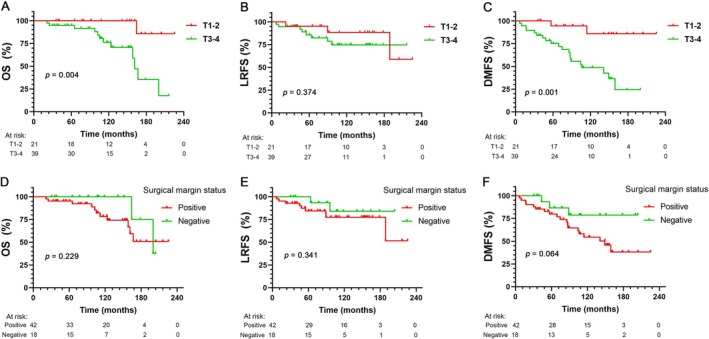
Kaplan–Meier curve of overall survival (OS) (A), local recurrence‐free survival (LRFS) (B) and distant metastasis‐free survival (DMFS) (C) by T stage; Kaplan–Meier estimates of OS (D), LRFS (E), and DMFS (F) by surgical margin status.

There was a significant difference in the estimated 10‐year OS rates between patients with early stages (T1–2) and advanced stages (T3–4) (100% vs. 76.1%, *p* = 0.004, Figure 3A); however, no difference in the estimated 10‐year LRFS rates was observed for patients with early stages and advanced stages (88.4% vs. 74.8%, *p* = 0.374, Figure 3B); the estimated 10‐year DMFS rates significantly increased with T stages (85.9% vs. 47.4%, *p* = 0.001, Figure 3C). In addition, compared to positive resection margins, negative surgical margins showed slight improvements in 10‐year OS (100% vs. 78.3%, *p* = 0.23, Figure 3D), LRFS (84.0% vs. 77.4%, *p* = 0.34, Figure 3E), and DMFS (78.8% vs. 53.3%, *p* = 0.064, Figure 3F), but without statistical difference. Univariate and multivariate Cox regression analyses were performed for OS and DMFS. Only the advanced T stage (T3–4 vs. T1–2) was identified as an independent unfavorable prognostic factor for both OS (*p* = 0.019) (Table 2) and DMFS (*p* = 0.006) (Table 3).

**TABLE 2:** Univariate and multivariate analysis of prognostic factors for overall survival.

Variables	Univariate analysis		Multivariate analysis	
	HR (95% CI)	*p*	HR (95% CI)	*p*
Age (≤ 50 y vs. > 50 y)	1.026 (0.343–3.071)	0.963	1.031 (0.320–3.323)	0.959
Gender (Male vs. Female)	0.692 (0.225–2.123)	0.519	0.337 (0.090–1.258)	0.106
Pittsburgh stage (T1–2 vs. T3–4)	11.338 (1.449–88.726)	0.021	15.193 (1.573–146.765)	0.019
Surgical margin status (Positive vs. Negative)	2.450 (0.542–11.086)	0.245	2.182 (0.383–12.441)	0.380
PNI (Yes vs. No)	2.488 (0.812–7.628)	0.111	1.914 (0.557–6.569)	0.302
RT technique (3D‐CRT vs. IMRT/VMAT)	3.643 (0.623–21.297)	0.151	3.556 (0.437–28.938)	0.236
ENI (Yes vs. No)	1.973 (0.651–5.985)	0.230	0.894 (0.264–3.031)	0.857
Chemotherapy (Yes vs. No)	1.689 (0.364–7.843)	0.504	0.998 (0.182–5.488)	0.998

Abbreviations: 3D‐CRT, three‐dimensional conformal radiotherapy; CI, confidence interval; ENI, elective neck irradiation; HR, hazard ratio; IMRT, intensity‐modulated radiotherapy; PNI, perineural invasion; RT, radiotherapy; VMAT, volumetric modulated arc therapy.

**TABLE 3:** Univariate and multivariate analysis of prognostic factors for distant metastasis‐free survival.

Variables	Univariate analysis		Multivariate analysis	
	HR (95% CI)	*p*	HR (95% CI)	*p*
Age (≤ 50 y vs. > 50 y)	1.420 (0.614–3.283)	0.412	1.204 (0.506–2.865)	0.676
Gender (Male vs. Female)	0.708 (0.306–1.636)	0.419	0.539 (0.219–1.326)	0.178
Pittsburgh stage (T1–2 vs. T3–4)	8.167 (1.900–35.116)	0.005	8.867 (1.890–41.587)	0.006
Surgical margin status (Positive vs. Negative)	2.980 (0.885–10.037)	0.078	2.098 (0.567–7.765)	0.267
PNI (Yes vs. No)	1.452 (0.624–3.378)	0.386	1.088 (0.429–2.756)	0.859
RT technique (3D‐CRT vs. IMRT/VMAT)	1.423 (0.459–4.408)	0.541	1.136 (0.329–3.919)	0.840
ENI (Yes vs. No)	1.721 (0.747–3.966)	0.202	1.000 (0.402–2.489)	0.999
Chemotherapy (Yes vs. No)	1.448 (0.489–4.289)	0.504	0.828 (0.263–2.604)	0.746

Abbreviations: 3D‐CRT, three‐dimensional conformal radiotherapy; CI, confidence interval; ENI, elective neck irradiation; HR, hazard ratio; IMRT, intensity‐modulated radiotherapy; PNI, perineural invasion; RT, radiotherapy; VMAT, volumetric modulated arc therapy.

### 3.3 ENI for cN0 Patients

**FIGURE 4:**
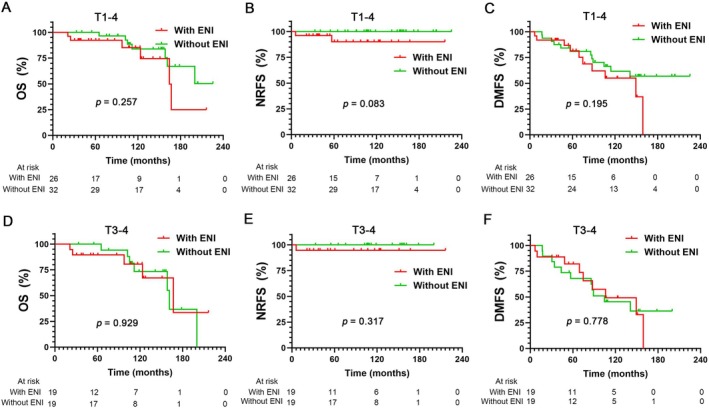
Kaplan–Meier estimates of overall survival (OS) (A), nodal recurrence‐free survival (NRFS) (B), and distant metastasis‐free survival (DMFS) (C) for ENI and without ENI in cN0 patients (T1–4); Kaplan–Meier estimates of OS (D), NRFS (E), and DMFS (F) for ENI and without ENI in cN0 patients (T3–4).

Among the 58 cN0 patients treated with PORT, 32 (55.2%) received radiation to the tumor bed alone, and 26 patients (44.8%) received ipsilateral ENI. The region of ENI for the 26 cN0 patients is shown in Figure S3. The 10‐year OS was 85.2% in the ENI group versus 83.8% for those without ENI (*p* = 0.257) (Figure 4A); similarly, there was no statistical difference in the 10‐year NRFS between the ENI group and the without ENI group (90.1% vs. 100%, *p* = 0.083) (Figure 4B). The 10‐year DMFS (*p* = 0.195) rates also showed no significant difference between these two groups (Figure 4C). Further analysis indicated that cN0 patients with T3–4 demonstrated no extended OS, NRFS, or DMFS underwent the ENI group compared with the patients without ENI (10‐year OS: 80.5% vs. 73.4%, *p* = 0.929; 10‐year NRFS: 94.7% vs. 100%, *p* = 0.317; 10‐year DMFS: 46.6% vs. 45.3%, *p* = 0.778) (Figure 4D–F).

### 3.4 Late Toxicities

Forty‐five patients were available for late toxicity evaluation. The results are depicted in Table 4. 73.3% (33/45) of patients complained of grade 3–4 (G3–4) ipsilateral hearing loss after surgery. The most common late toxicities related to RT were xerostomia (31.1%, 14/45) and radiation‐related caries (26.7%, 12/45). Seven patients had G3 radiation‐related caries, two patients had G3 xerostomia, and one patient had G3 trismus. Additionally, three patients experienced impairment of masticatory function. No patient experienced a fatal event.

**TABLE 4:** Treatment‐related adverse events of 45 patients.

Adverse event type	Any G *n* (%)	G1 *n* (%)	G2 *n* (%)	G3 *n* (%)	G4 *n* (%)
Ipsilateral hearing loss	34 (75.6)	1 (2.2)	0	2 (4.4)	31 (68.9)
Tinnitus	5 (11.1)	3 (6.7)	2 (4.4)	0	0
Otalgia	3 (6.7)	3 (6.7)	0	0	0
Xerostomia	14 (31.1)	8 (17.8)	4 (8.9)	2 (4.4)	0
Trismus	2 (4.4)	1 (2.2)	0	1 (2.2)	0
Radiation‐related caries	12 (26.7)	2 (4.4)	3 (6.7)	7 (15.6)	0
Dysphagia	2 (4.4)	2 (4.4)	0	0	0

Abbreviation: G, Grade; No patient occurred fatal event (G5).

## 4 Discussion

Resection with clear margins is the mainstay treatment strategy for ACC, and postoperative radiotherapy (PORT) could reduce local recurrence. However, whether PORT could extend survival is still controversial, and high‐quality, evidence‐based reports have yet to be made available. This study investigated the impact of RT specifically on EAC ACC. Our study found that surgery plus PORT achieved satisfactory long‐term survival for patients with this rare disease. The 10‐year OS, LRFS, NRFS, and DMFS rates were 84.2%, 79.5%, 96.2%, and 59.9%, respectively, which were significantly higher than those in prior studies [4, 10, 14].

The clinical course of ACC is characterized by a high rate of local recurrence (LR) [15]. In the study by Chen et al. [4], LR occurred in 33% of patients with EAC ACC after surgery. The high LR rate might be due to a strong tendency for perineural invasion (48%–82%) and the proximity to vital structures, making it difficult to achieve negative margins [16]. Several studies identified positive margins as an independent negative prognostic factor in patients with head and neck ACCs [17, 18]. Achieving clear margins/R0 resection during the initial surgery is the treatment goal. In the present study, 70% of patients with EAC ACC had positive surgical margins, largely due to the high proportion of advanced‐stage patients (T3–4, 65%) and the complex anatomical structures of the aural region and temporal region. However, contrary to previous studies, margin status did not statistically affect the OS (*p* = 0.229), LRFS (*p* = 0.341), and DMFS (*p* = 0.064) in patients with EAC ACC who underwent PORT in our analysis. A possible explanation for this difference might be that radiation therapy plays a vital role as a complementary treatment for surgery in lesions with positive margins. Furthermore, our data showed only 11 patients (18.3%) experienced LR after PORT, which was significantly lower than the previous report (33%), possibly attributed to the application of RT [4]. Irradiation coverage could track the perineural tract to reduce the chance of tumor dissemination along the peri‐nerve.

A high tendency for distant metastasis (DM) is another common malignant feature of ACC. Previous literature suggested that DM is a poor prognostic factor for patients with EAC ACC [7]. A notable finding in our study was that DM was the predominant pattern of failure for EAC ACC after RT. Twenty‐three patients (38.3%) developed DM during the long‐term follow‐up, including 21 cases with lung metastasis, which is in line with the results of previous studies [19]. Moreover, we found that DM was the primary cause of death for EAC ACC patients (76.9%, 10/13). Therefore, preventing metastasis seems crucial to ensure adequate survival and to reduce morbidity. The treatment of DM has always been challenging in the management of ACC due to the low response rate to chemotherapy and molecular‐targeted therapies [12]. Screening for out the risk factors related to DM and intervening early can effectively reduce the probability of DM and thus improve outcomes. Increasing evidence indicates that patients with a solid subtype of histopathology are associated with the poorest prognosis and a clear tendency to DM, which needs more active adjuvant treatment and close follow‐up [19, 20, 21]. Dai et al. suggested that advanced T stage is related to increased DM risk and that parotid gland invasion was associated with worse DMFS [19, 22]. Similarly, our study demonstrated that patients with an advanced T stage (T3–4) had a higher risk of DM and poorer OS; therefore, early diagnosis and efficient treatment could significantly improve the prognosis. At present, DM remains the most difficult treatment challenge, and the mechanisms underlying the high risk of DM for EAC ACC patients require further investigation. Although patients with DM are almost incurable, prolonged survival is possible. The role of targeted therapy, immunotherapy, or other adjuvant systemic treatments in patients at high risk of DM needs, therefore, to be further investigated in prospective trials.

Whether ENI is necessary for the EAC ACC is still a matter of great debate. Node metastasis is rare in the head and neck area of ACC [23, 24]. In the current study, only 2 (3.3%) patients presented with node metastasis at presentation, and 2 (3.3%) experienced a regional nodal relapse. Current data on EAC ACC were insufficient to determine whether ENI should be delivered to cN0 patients. Our study found no difference in survival outcomes among patients who received ENI compared with those who did not, regardless of the T‐stage. Therefore, the ENI for cN0 patients appears unnecessary, as it offers no outcome benefit. The omission of ENI may significantly reduce RT‐related toxicity (e.g., dermatitis, hypothyroidism, and late subcutaneous fibrosis) and thus improve the patient's quality of life.

EAC ACC is a rare head and neck malignancy with limited recent studies to define expected late toxicity after radiotherapy. In our study, grade 3–4 ipsilateral hearing loss (73.3%, 33/45) was the most common complication after surgery. However, since contralateral hearing is normal, it has little impact on patients' daily lives. Furthermore, xerostomia (31.1%, 14/45) and radiation‐related caries (26.7%, 12/45) were the most common late toxicities after PORT, and no patients developed osteoradionecrosis (ORN), which had been reported previously in EAC tumors [25]. In sum, our study showed that PORT was effective and safe for EAC ACC. Chen et al. reported that IMRT offered better target coverage and lower treatment‐related toxicity in the squamous cell carcinoma (SCC) of the EAC and middle ear; therefore, IMRT might be the preferred modality for RT due to the lower late toxicity [26].

This study has several limitations. First, the research adopted a single‐center retrospective design, which inherently carries risks of selection bias (due to non‐random patient enrollment) and information bias (due to reliance on historical medical records with potential variability in data documentation). Second, the relatively small sample size may limit the statistical power of our findings, particularly when interpreting subgroup trends or rare outcomes. Third, we lacked detailed information on histopathological subtypes (e.g., tubular, cribriform, or solid subtypes) of the disease. Despite these limitations, to our knowledge, this is one of the largest reported series focused on the role of radiotherapy for EAC ACC.

In conclusion, surgery plus PORT is associated with excellent long‐term survival with tolerable late toxicity for patients with EAC ACC. Ipsilateral ENI of cN0 patients shows no survival benefit, which suggests that the omission of ENI is reasonable to reduce radiation‐related toxicity. Despite the excellent survival rates, the DM rate remains high and occurs later after PORT, highlighting the importance of continuous research and collaborative clinical efforts.

## Author Contributions

Li Wang and Xiaoshen Wang made substantial contributions to the conception or design of the work; Haojiong Zhang, Zi‐Wei Tu, Tianci Tang, and Gang Yang are responsible for administrative/technical/material support and study supervision; Statistical analysis was undertaken by Li Wang, Xinmao Song, and Zhenhu Li; Li Wang, Xiaoshen Wang, and Xinmao Song drafted the article and reviewed the submitted version of the manuscript; Xinmao Song, Zhenhu Li, Lifen Zou, and Zi‐Wei Tu helped out with the results discussion.

## Funding

This project was supported financially by grants from the Natural Science Foundation of China (82103059), the Excellent Young Scientists Fund of Jiangxi Cancer Hospital (2021EYS03), and Fudan University Affiliated Jinshan Hospital youth Research Fund (JYQN‐202302).

## Ethics Statement

The Clinical Research Ethics Committee of the Eye, Ear, Nose & Throat Hospital of Fudan University approved this study (2021033‐1), and all the patients provided written informed consent before treatment.

## Conflicts of Interest

The authors declare no conflicts of interest.

## Supporting information


**Figure S1:** Treatment group schemes. Flowchart describing definitive treatment disposition. 3D‐CRT, three‐dimensional conformal radiotherapy; IMRT, intensity‐modulated radiation therapy; PORT, postoperative radiotherapy.

**Figure S2:** Findings for a 41‐year‐old man with T3 EAC ACC. (a, b) Preoperative ear T1‐weighted gadolinium‐enhanced MRI image (The white arrow depicts lesion); (c, d) Postoperative ear T1‐weighted gadolinium‐enhanced MRI image; the dose distribution of IMRT (e) axial, (f) coronal, and (g) sagittal for the same patient. IMRT, intensity‐modulated radiation therapy; MRI, magnetic resonance imaging.

**Figure S3:** Axial (a) and coronal (b) view T1‐weighted gadolinium‐enhanced MRI of an EAC ACC patient (case 1) with brain metastasis; Axial (c) and coronal (d) display T1‐weighted gadolinium‐enhanced MRI of another EAC ACC patient (case 2) with local recurrence; Axial (e) showed lung metastasis for the same patient (case 2).

**Figure S4:** The region of ENI for the 26 cN0 patients.

**Figure S5:** Cumulative incidence of local recurrence stratified by T stage (A), surgical margin status (C) and ENI (E); Cumulative incidence of distant metastasis stratified by T stage (B), surgical margin status (D) and ENI (F). LRFS, local recurrence‐free survival; DMFS, distant metastasis‐free survival; ENI, elective neck irradiation.

**Table S1:** Symptoms and signs of 60 patients with ACC of the EAC.

**Table S2:** Clinical features and death analysis of 13 patients.

## Data Availability

Research data are stored in an institutional repository and will be shared upon request to the corresponding author.

## References

[1] G. Cantù , “Adenoid Cystic Carcinoma. An Indolent but Aggressive Tumour. Part B: Treatment and Prognosis,” Acta Otorhinolaryngologica Italica 41, no. 4 (2021): 296–307. 34533533 10.14639/0392-100X-N1729 PMC8448184

[2] A. Coca‐Pelaz , J. P. Rodrigo , P. J. Bradley , et al., “Adenoid Cystic Carcinoma of the Head and Neck—An Update,” Oral Oncology 51, no. 7 (2015): 652–661. 25943783 10.1016/j.oraloncology.2015.04.005

[3] T. Zhang , C. F. Dai , and Z. M. Wang , “The Misdiagnosis of External Auditory Canal Carcinoma,” European Archives of Oto‐Rhino‐Laryngology 270, no. 5 (2013): 1607–1613. 22926989 10.1007/s00405-012-2159-4

[4] S. L. Chen , S. F. Huang , V. Wai‐Yee Ho , V. W.‐Y. Ho , W.‐Y. Chuang , and K.‐C. Chan , “Clinical Characteristics and Treatment Outcome of Adenoid Cystic Carcinoma in the External Auditory Canal,” Biomedical Journal 43, no. 2 (2020): 189–194. 32389593 10.1016/j.bj.2019.07.005 PMC7283548

[5] P. J. Bradley , “Adenoid Cystic Carcinoma Evaluation and Management: Progress With Optimism!,” Current Opinion in Otolaryngology & Head and Neck Surgery 25, no. 2 (2017): 147–153. 28106659 10.1097/MOO.0000000000000347

[6] A. B. Vardag , M. H. Danish , M. S. Awan , M. U. Tariq , O. A. Bhatti , and M. Hammad , “Adenoid Cystic Carcinoma of External Auditory Canal: A Rare Disease,” Journal of the Pakistan Medical Association 71, no. 7 (2021): 1893–1896. 34410269 10.47391/JPMA.572

[7] R. W. Green and U. C. Megwalu , “Adenoid Cystic Carcinoma of the External Ear: A Population Based Study,” American Journal of Otolaryngology 37, no. 7 (2016): 346–350. 27040415 10.1016/j.amjoto.2016.02.001

[8] R. W. Gao , D. M. Routman , W. S. Harmsen , et al., “Adenoid Cystic Carcinoma of the Head and Neck: Patterns of Recurrence and Implications for Intensity‐Modulated Radiotherapy,” Head & Neck 45, no. 1 (2023): 187–196. 36222355 10.1002/hed.27223

[9] S. H. Choi , A. J. Yang , S. O. Yoon , et al., “Role of Postoperative Radiotherapy in Resected Adenoid Cystic Carcinoma of the Head and Neck,” Radiation Oncology 17, no. 1 (2022): 197. 36456955 10.1186/s13014-022-02165-5 PMC9716721

[10] K. Hayashi , M. Koto , Y. Demizu , et al., “A Retrospective Multicenter Study of Carbon‐Ion Radiotherapy for External Auditory Canal and Middle Ear Carcinomas,” Cancer Medicine 8, no. 1 (2019): 51–57. 30548207 10.1002/cam4.1830 PMC6346229

[11] A. Al‐Mamgani , P. van Rooij , A. Sewnaik , L. Tans , and J. A. Hardillo , “Adenoid Cystic Carcinoma of Parotid Gland Treated With Surgery and Radiotherapy: Long‐Term Outcomes, QoL Assessment and Review of the Literature,” Oral Oncology 48, no. 3 (2012): 278–283. 22093375 10.1016/j.oraloncology.2011.10.014

[12] G. Papaspyrou , S. Hoch , A. Rinaldo , et al., “Chemotherapy and Targeted Therapy in Adenoid Cystic Carcinoma of the Head and Neck: A Review,” Head & Neck 33, no. 6 (2011): 905–911. 20652885 10.1002/hed.21458

[13] S. Morita , T. Mizumachi , Y. Nakamaru , et al., “Comparison of the University of Pittsburgh Staging System and the Eighth Edition of the American Joint Committee on Cancer TNM Classification for the Prognostic Evaluation of External Auditory Canal Cancer,” International Journal of Clinical Oncology 23, no. 6 (2018): 1029–1037. 29974295 10.1007/s10147-018-1314-3

[14] F. M. Gu , F. L. Chi , C. F. Dai , B. Chen , and H. W. Li , “Surgical Outcomes of 43 Cases With Adenoid Cystic Carcinoma of the External Auditory Canal,” American Journal of Otolaryngology 34, no. 5 (2013): 394–398. 23453117 10.1016/j.amjoto.2013.01.018

[15] G. Cantù , “Adenoid Cystic Carcinoma. An Indolent but Aggressive Tumour. Part A: From Aetiopathogenesis to Diagnosis,” Acta Otorhinolaryngologica Italica 41, no. 3 (2021): 206–214. 34264913 10.14639/0392-100X-N1379 PMC8283400

[16] Y. Fang , Z. Y. Peng , Y. M. Wang , et al., “Current Opinions on Diagnosis and Treatment of Adenoid Cystic Carcinoma,” Oral Oncology 130 (2022): 105945. 35662026 10.1016/j.oraloncology.2022.105945

[17] M. Amit , S. Na'ara , L. Trejo‐Leider , et al., “Defining the Surgical Margins of Adenoid Cystic Carcinoma and Their Impact on Outcome: An International Collaborative Study,” Head & Neck 39, no. 5 (2017): 1008–1014. 28252829 10.1002/hed.24740 PMC5879774

[18] S. Takebayashi , S. Shinohara , H. Tamaki , et al., “Adenoid Cystic Carcinoma of the Head and Neck: A Retrospective Multicenter Study,” Acta Oto‐Laryngologica 138, no. 1 (2018): 73–79. 28899226 10.1080/00016489.2017.1371329

[19] Y. B. Zhang , H. Y. Liu , Q. R. Wu , S. Wang , and C. Dai , “Predictors of Distant Metastasis and Survival in Adenoid Cystic Carcinoma of the External Auditory Canal,” Otology & Neurotology 40, no. 10 (2019): e1006–e1011. 31688611 10.1097/MAO.0000000000002391

[20] J. Y. Lee and Y. S. Cho , “Clinical Outcome and Prognostic Factors in Adenoid Cystic Carcinoma of the External Auditory Canal: Proposal for a Refined T‐Stage Classification System,” European Archives of Oto‐Rhino‐Laryngology 280, no. 8 (2023): 3625–3633. 36781438 10.1007/s00405-023-07876-3

[21] H. Y. Liu , Y. B. Zhang , T. Zhang , F. Li , and C. Dai , “Correlation Between the Pathology and Clinical Presentations in Patients With Adenoid Cystic Carcinoma of the External Auditory Canal,” Head & Neck 39, no. 12 (2017): 2444–2449. 28963786 10.1002/hed.24915

[22] F. T. Li , J. Wang , Y. S. Feng , et al., “The Role of Parotid Gland Invasion in Adenoid Cystic Carcinoma of the External Auditory Canal,” Laryngoscope 134, no. 1 (2024): 419–425. 37421252 10.1002/lary.30855

[23] International Head and Neck Scientific Group , “Cervical Lymph Node Metastasis in Adenoid Cystic Carcinoma of the Sinonasal Tract, Nasopharynx, Lacrimal Glands and External Auditory Canal: A Collective International Review,” Journal of Laryngology and Otology 130, no. 12 (2016): 1093–1097. 27839526 10.1017/S0022215116009373 PMC5535774

[24] S. Atallah , A. Moya‐Plana , O. Malard , et al., “Should a Neck Dissection Be Performed on Patients With cN0 Adenoid Cystic Carcinoma? A REFCOR Propensity Score Matching Study,” European Journal of Cancer 130 (2020): 250–258. 32008920 10.1016/j.ejca.2019.12.026

[25] S. G. Laskar , S. Sinha , P. Pai , et al., “Definitive and Adjuvant Radiation Therapy for External Auditory Canal and Temporal Bone Squamous Cell Carcinomas: Long Term Outcomes,” Radiotherapy and Oncology 170 (2022): 151–158. 35219800 10.1016/j.radonc.2022.02.021

[26] W. Y. Chen , S. H. Kuo , Y. H. Chen , et al., “Postoperative Intensity‐Modulated Radiotherapy for Squamous Cell Carcinoma of the External Auditory Canal and Middle Ear: Treatment Outcomes, Marginal Misses, and Perspective on Target Delineation,” International Journal of Radiation Oncology, Biology, Physics 82, no. 4 (2012): 1485–1493. 21775071 10.1016/j.ijrobp.2011.05.018

