# Supplementary figures and images for: Survival Outcomes and Late Toxicity of Postoperative Radiotherapy in Patients With Adenoid Cystic Carcinoma of the External Auditory Canal
Source: Cancer Med. 2025 Dec 29;15(1):e71501. doi: 10.1002/cam4.71501 (PMC12745885; doi:10.1002/cam4.71501)

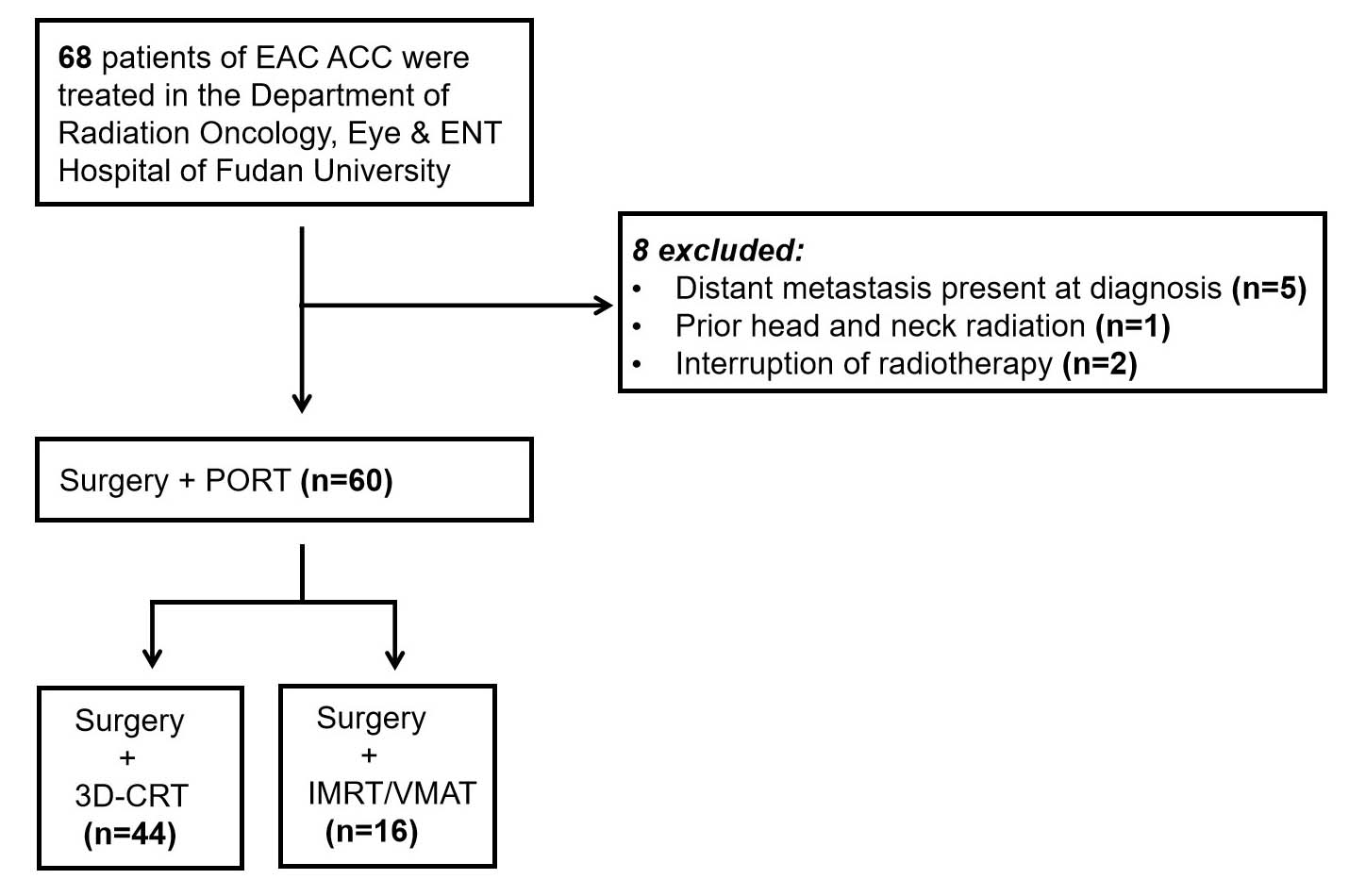

Supplement: Supplementary file 1 — Figure S1: Treatment group schemes. Flowchart describing definitive treatment disposition. 3D‐CRT, three‐dimensional conformal radiotherapy; IMRT, intensity‐modulated radiation therapy; PORT, postoperative radiotherapy. [file CAM4-15-e71501-s002.jpg]

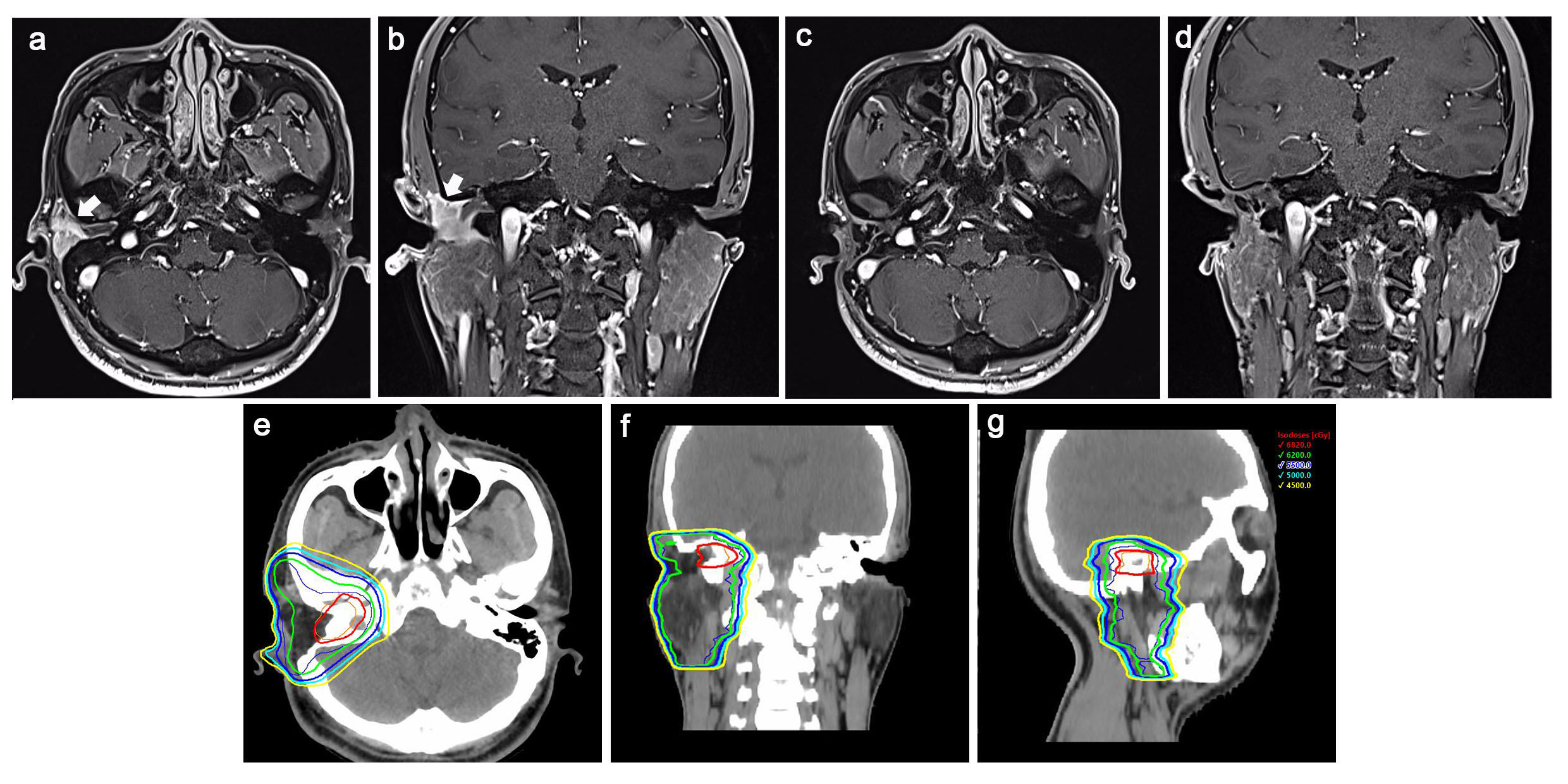

Supplement: Supplementary file 2 — Figure S2: Findings for a 41‐year‐old man with T3 EAC ACC. (a, b) Preoperative ear T1‐weighted gadolinium‐enhanced MRI image (The white arrow depicts lesion); (c, d) Postoperative ear T1‐weighted gadolinium‐enhanced MRI image; the dose distribution of IMRT (e) axial, (f) coronal, and (g) sagittal for the same patient. IMRT, intensity‐modulated radiation therapy; MRI, magnetic resonance imaging. [file CAM4-15-e71501-s003.jpg]

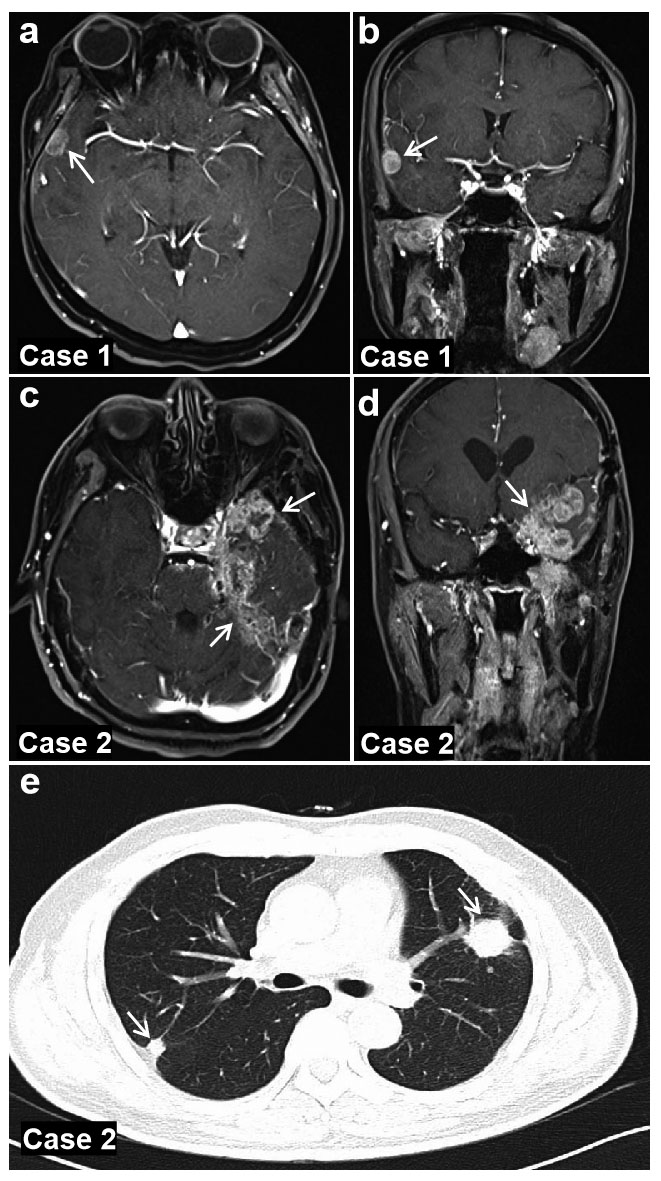

Supplement: Supplementary file 3 — Figure S3: Axial (a) and coronal (b) view T1‐weighted gadolinium‐enhanced MRI of an EAC ACC patient (case 1) with brain metastasis; Axial (c) and coronal (d) display T1‐weighted gadolinium‐enhanced MRI of another EAC ACC patient (case 2) with local recurrence; Axial (e) showed lung metastasis for the same patient (case 2). [file CAM4-15-e71501-s005.jpg]

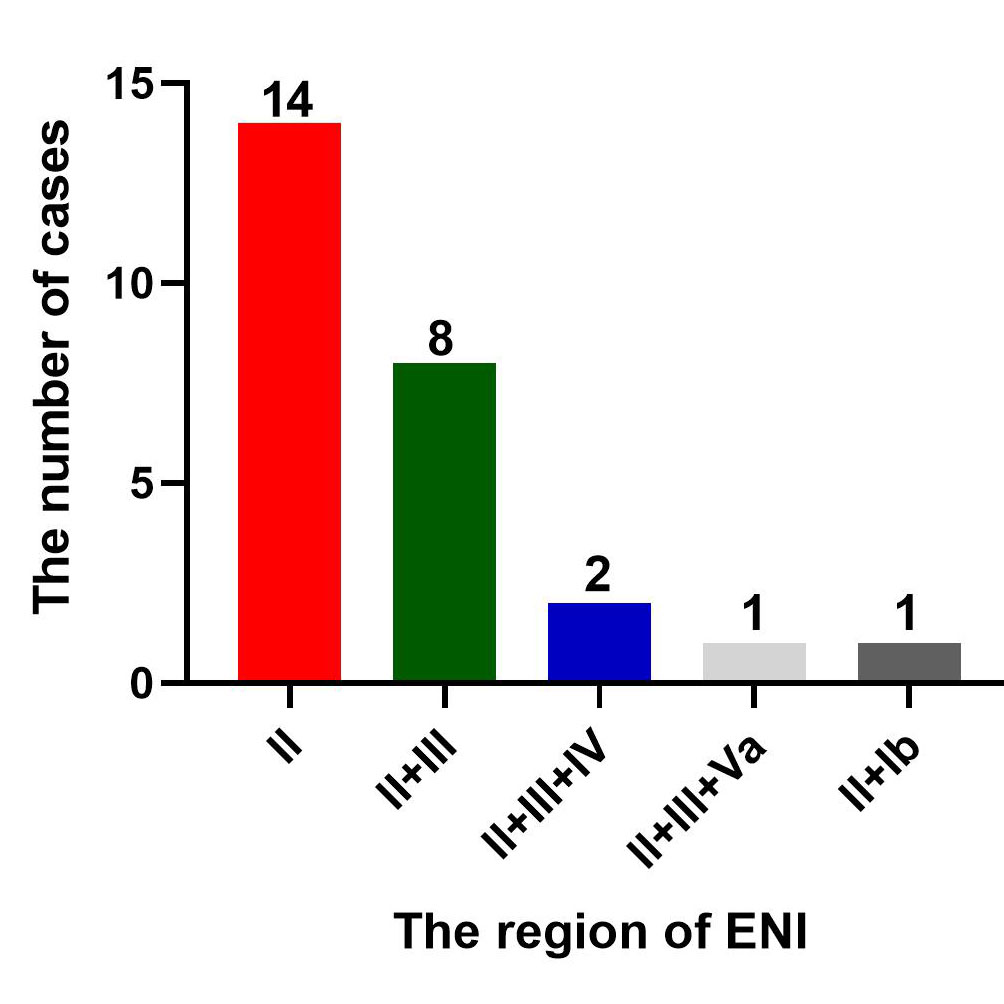

Supplement: Supplementary file 4 — Figure S4: The region of ENI for the 26 cN0 patients. [file CAM4-15-e71501-s007.jpg]

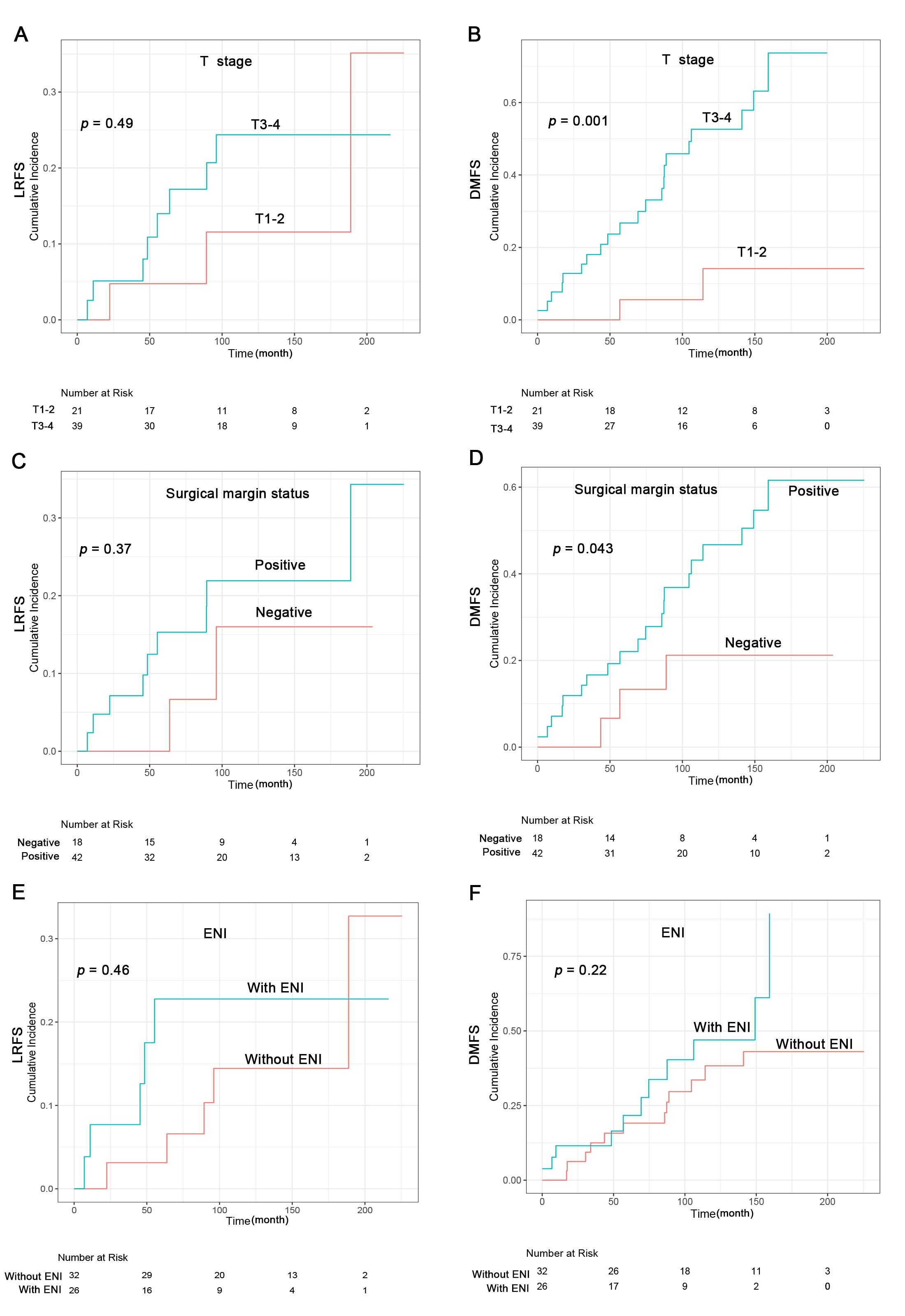

Supplement: Supplementary file 5 — Figure S5: Cumulative incidence of local recurrence stratified by T stage (A), surgical margin status (C) and ENI (E); Cumulative incidence of distant metastasis stratified by T stage (B), surgical margin status (D) and ENI (F). LRFS, local recurrence‐free survival; DMFS, distant metastasis‐free survival; ENI, elective neck irradiation. [file CAM4-15-e71501-s004.jpg]
