# Supplementary material for: Survival Outcomes and Late Toxicity of Postoperative Radiotherapy in Patients With Adenoid Cystic Carcinoma of the External Auditory Canal
Source: Cancer Med. 2025 Dec 29;15(1):e71501. doi: 10.1002/cam4.71501 (PMC12745885; doi:10.1002/cam4.71501)
Supplement: Supplementary file 6 — Table S1: Symptoms and signs of 60 patients with ACC of the EAC. [file CAM4-15-e71501-s001.docx]

| **Supplementary table 1. Symptoms and signs of 60 patients with ACC of the EAC.** | | |
| --- | --- | --- |
| **Symptoms and Signs** | **No. of Patients** | **%** |
| Otalgia | 43 | 71.7 |
| EAC Mass | 33 | 55.0 |
| Hearing loss | 8 | 13.3 |
| Otorrhea | 7 | 11.7 |
| Ear stuffy | 4 | 6.7 |
| Facial palsy | 4 | 6.7 |
