# Supplementary material for: Survival Outcomes and Late Toxicity of Postoperative Radiotherapy in Patients With Adenoid Cystic Carcinoma of the External Auditory Canal
Source: Cancer Med. 2025 Dec 29;15(1):e71501. doi: 10.1002/cam4.71501 (PMC12745885; doi:10.1002/cam4.71501)
Supplement: Supplementary file 7 — Table S2: Clinical features and death analysis of 13 patients. [file CAM4-15-e71501-s006.docx]

| Patient | Age | T stage | PNI | Surgical margin | | ENI | CT | Cause of death |
| --- | --- | --- | --- | --- | --- | --- | --- | --- |
| Case 1 | 65 | 4 | No | | Positive | No | Yes | Local recurrence |
| Case 2 | 49 | 3 | Yes | | Negative | No | No | Local recurrence |
| Case 3 | 59 | 3 | Yes | | Positive | Yes | No | Local recurrence |
| Case 4 | 53 | 4 | Yes | | Positive | Yes | Yes | Lung metastasis |
| Case 5 | 40 | 3 | No | | Positive | Yes | No | Bone metastasis |
| Case 6 | 38 | 4 | Yes | | Positive | No | No | Lung metastasis |
| Case 7 | 39 | 1 | No | | Negative | Yes | No | Bone metastasis |
| Case 8 | 44 | 3 | No | | Positive | Yes | No | Multiple sites^a^ |
| Case 9 | 29 | 3 | Yes | | Positive | No | No | Lung metastasis |
| Case 10 | 57 | 4 | Yes | | Positive | No | No | Lung metastasis |
| Case 11 | 62 | 4 | No | | Positive | No | No | Lung metastasis |
| Case 12 | 50 | 4 | No | | Positive | Yes | No | Lung metastasis |
| Case 13 | 52 | 4 | Yes | | Positive | No | No | Lung metastasis |

**Supplementary table 2. Clinical features and death analysis of 13 patients**

PNI, perineural Invasion; ENI, elective nodal irradiation; CT, chemotherapy;

Multiple sites^a^, Multiple sites metastasis included lung, liver, and kidney.
